# Acute psychiatric symptoms in a young woman with anti‐N‐methyl D‐aspartate receptor encephalitis: A case of successful early diagnosis and therapeutic intervention

**DOI:** 10.1002/ccr3.5420

**Published:** 2022-02-07

**Authors:** Marie Tominaga, Kyoko Morikawa, Hiroto Yamamoto, Yutaro Ogawa, Naomi Kamimura, Ikunosuke Tsuneki, Tetsuro Yahata, Masaki Tamura, Toru Yanase, Aki Sato, Hiroyuki Shibuya, Takumi Kurabayashi

**Affiliations:** ^1^ 26834 Department of Obstetrics & Gynecology Niigata City General Hospital Niigata Japan; ^2^ 26834 Department of Neurology Niigata City General Hospital Niigata Japan; ^3^ 26834 Department of Pathology Niigata City General Hospital Niigata Japan

**Keywords:** anti‐N‐methyl D‐aspartate receptor, autoimmune encephalitis, gynecology, laparoscopy, mature teratoma, neurology

## Abstract

This clinical image presents a report on the diagnosis and treatment of anti‐NMDAR encephalitis, a rare disease. This report emphasizes the importance of a differential diagnosis for acute psychiatric symptoms. Accurate and timely diagnosis is critical for the selection and implementation of treatment and for optimal patient outcomes.

A 22‐year‐old woman acutely developed abnormal speech without prodromal symptoms. On the following day, she stabbed herself in the left chest with a knife and was rushed to our hospital. Her family and medical history were unremarkable. She was diagnosed with hemopneumothorax on chest radiography and computed tomography (CT) (Figure 1), prompting her to undergo thoracocentesis. She then developed bilateral upper extremity tonic spasms and orofacial dyskinesia. Head magnetic resonance imaging (MRI) revealed no abnormalities, and there was no increase in her cerebrospinal fluid (CSF) cell count. Electroencephalography demonstrated extreme delta brushes with high amplitude 3 Hz rhythmic slowing and superimposed low amplitude 15–20 Hz fast waves. Pelvic CT revealed a tumor in the right ovary (Figure 2).

**FIGURE 1:**
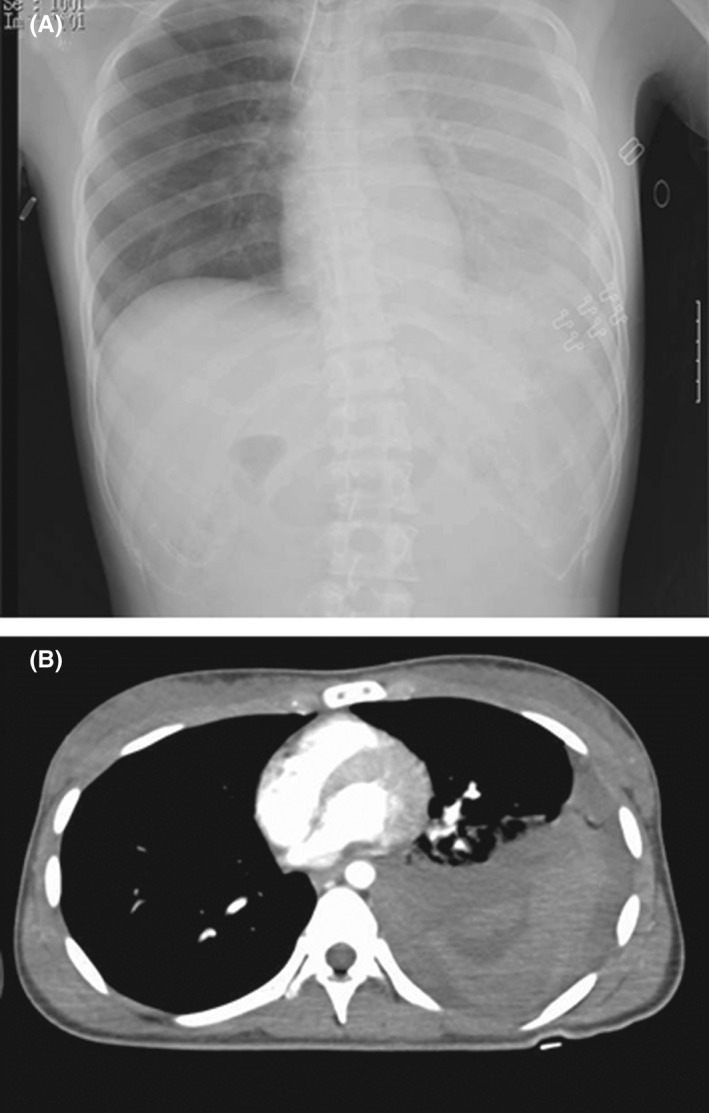
(A) Chest radiography and (B) computed tomography (CT) showing pleural effusion in the left lung

**FIGURE 2:**
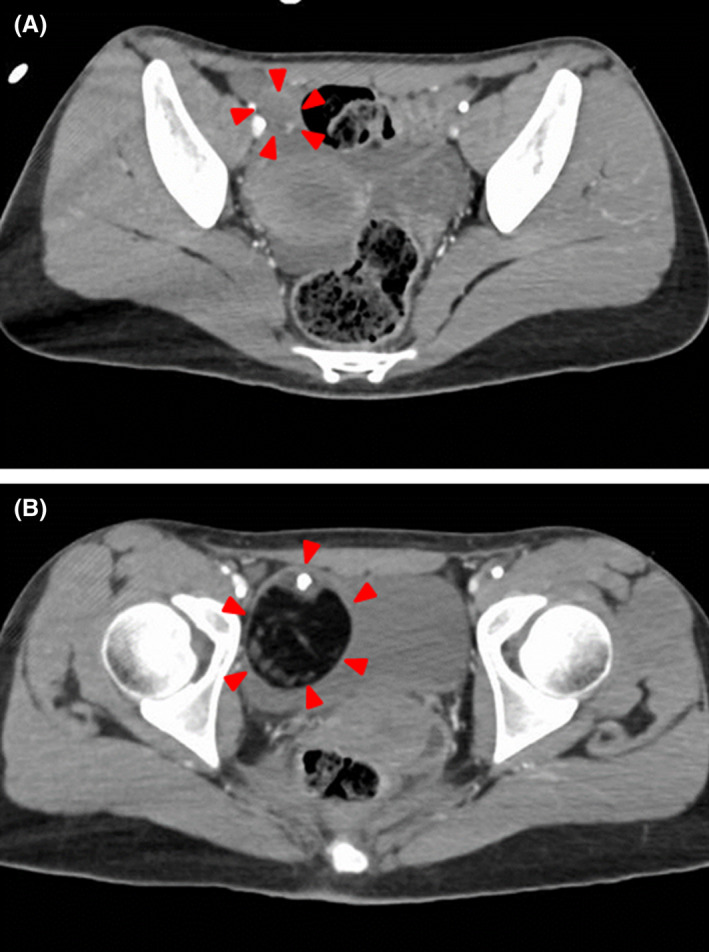
(A) and (B) Pelvic CT showing a tumor with internal calcification in the right ovary (red arrowhead)

She was diagnosed with anti‐N‐methyl D‐aspartate receptor (NMDAR) encephalitis based on her positive anti‐NMDAR antibodies detected on CSF analysis (CSF analysis was outsourced). Ten days after her symptom onset, laparoscopic right adnexal resection was performed (Figure 3), resulting in a pathological diagnosis of ovarian teratoma (Figure 4). She was treated with additional steroid pulses, high‐dose immunoglobulin therapy, and plasma exchange. The symptoms were relieved in approximately 45 days post‐surgery, and no relapse was noted.

**FIGURE 3:**
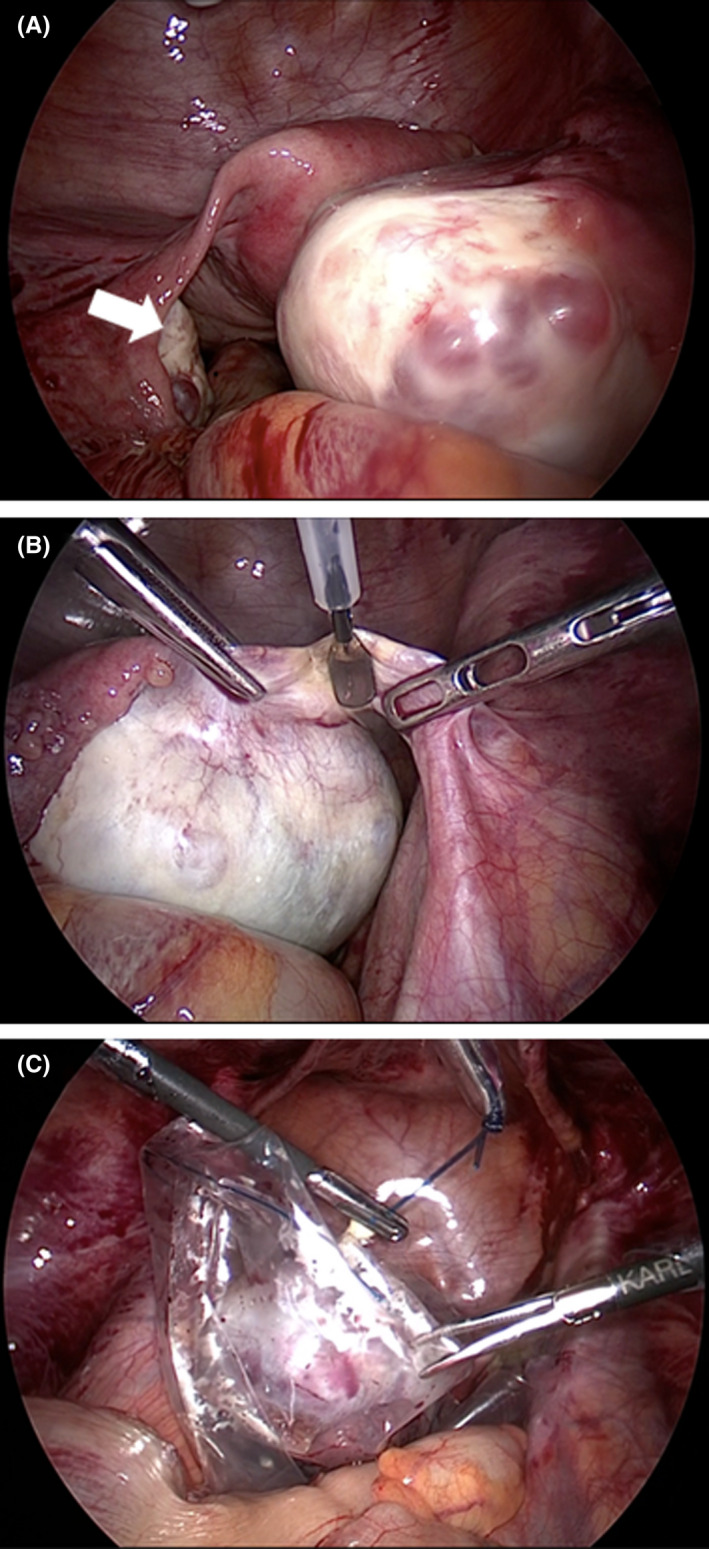
Intraoperative photographs. (A) Right ovary is enlarged, and the left adnexa (white arrow) shows no abnormal findings grossly. (B) The right adnexa is resected laparoscopically. (C) The tumor specimen is collected in a tissue collection bag. Care was taken to prevent leakage of tumor contents into the abdominal cavity

**FIGURE 4:**
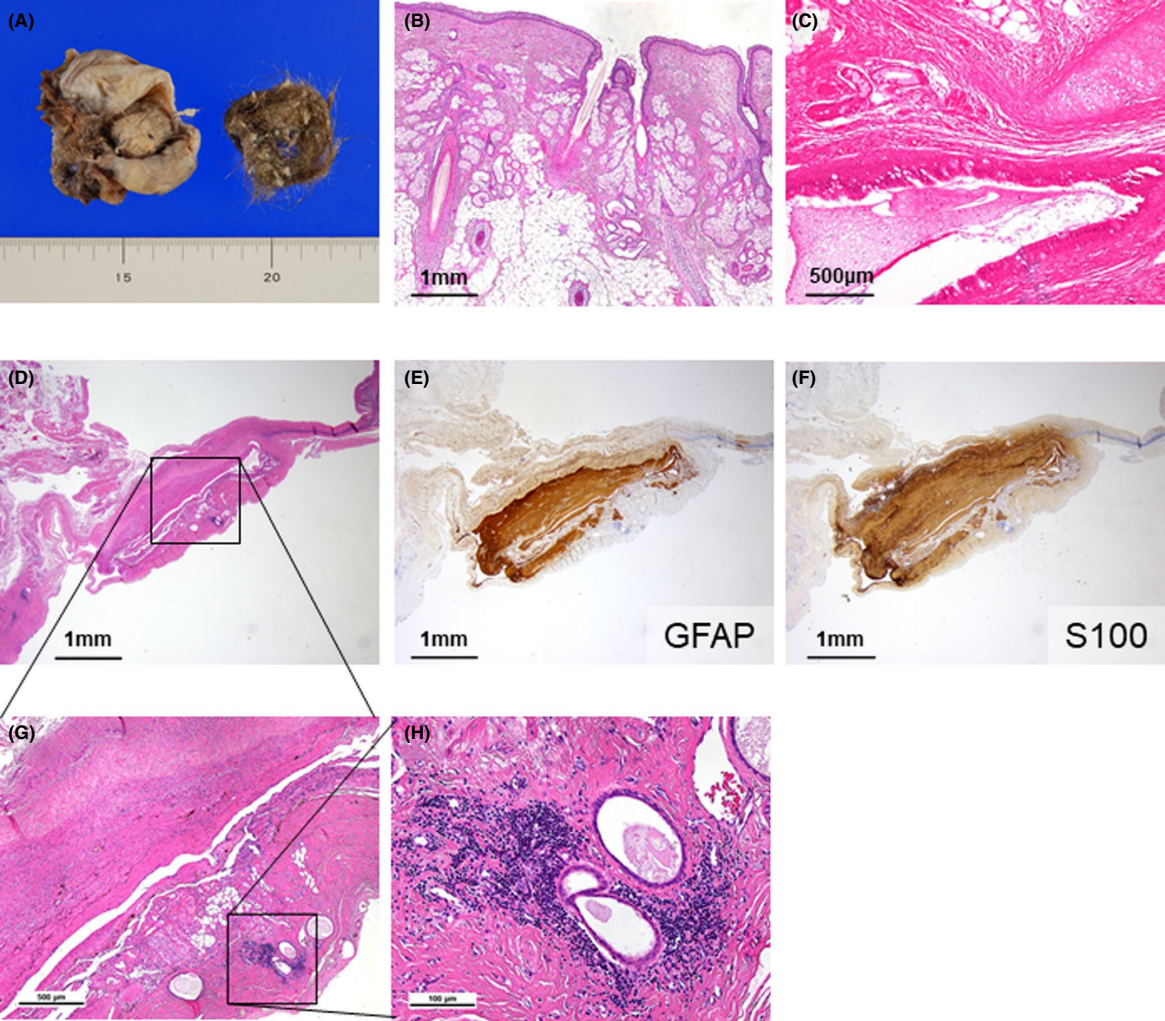
Macroscopic and pathological findings. (A) Macroscopic findings of the ovarian tumor: 5 cm in size, with hair (B). (C) Pathological findings showing tissue of skin appendages and bronchial tissue. (D–H) Pathological findings showing positive neurological markers on immunohistochemical staining and lymphocytic infiltration. No malignant findings were noted on pathological examination

Anti‐NMDAR encephalitis is a paraneoplastic, immune‐mediated encephalopathy associated with ovarian teratomas.^1^ Recovery is typically slow and erratic and may take >3 years.^2^ This report emphasizes the importance of accurate and timely diagnosis and treatment, resulting in quick recovery with good prognosis.

## CONFLICT OF INTEREST

The authors declare that they have no current financial arrangements or affiliations with any organization that may have a direct influence on their work.

## AUTHOR CONTRIBUTIONS

All the authors made substantial contribution to the preparation of this manuscript and approved the final version for submission. MT, KM, HY, and YO drafted the manuscript. MT involved in corresponding author. NK, IT, TY, MT, TY, AS, and HS involved in clinical support. TK involved in review of the manuscript carefully.

## CONSENT

Written informed consent was obtained from the patients for the publication of their information and imaging findings.

## Data Availability

Not Applicable.
